# Identification of Bacteria Associated with Post-Operative Wounds of Patients with the Use of Matrix-Assisted Laser Desorption/Ionization Time-of-Flight Mass Spectrometry Approach

**DOI:** 10.3390/molecules26165007

**Published:** 2021-08-18

**Authors:** Małgorzata Szultka-Młyńska, Daria Janiszewska, Paweł Pomastowski, Michał Złoch, Wojciech Kupczyk, Bogusław Buszewski

**Affiliations:** 1 Department of Environmental Chemistry and Bioanalytics, Faculty of Chemistry, Nicolaus Copernicus University, Gagarin 7, 87-100 Torun, Poland; janiszewska_daria@doktorant.umk.pl (D.J.); bbusz@umk.pl (B.B.); 2 Centre for Modern Interdisciplinary Technologies, Nicolaus Copernicus University, Wilenska 4, 87-100 Torun, Poland; pawel_pomastowski@wp.pl (P.P.); michalzloch87@gmail.com (M.Z.); 3 Department of General, Gastroenterological and Oncological Surgery, Collegium Medicum, Nicolaus Copernicus University, 87-100 Torun, Poland; kupczykwojciech@tlen.pl

**Keywords:** antibiotic influence, bacterial strain, MALDI-TOF MS, post-operative wound

## Abstract

The bacterial infection of post-operative wounds is a common health problem. Therefore, it is important to investigate fast and accurate methods of identifying bacteria in clinical samples. The aim of the study was to analyse the use of the MALDI-TOF MS technique to identify microorganism wounds that are difficult to heal. The most common bacteria are *Escherichia coli*, *Staphylococcus* spp., and *Enterococcus* spp. We also demonstrate the effect of culture conditions, such as the used growth medium (solid: Brain Heart Infusion Agar, Mueller Hilton Agar, Glucose Bromocresol Purple Agar, and Vancomycin Resistance Enterococci Agar Base and liquid: Tryptic Soy Broth and BACTEC Lytic/10 Anaerobic/F), the incubation time (4, 6, and 24h), and the method of the preparation of bacterial protein extracts (the standard method based on the Bruker guideline, the Sepsityper method) to identify factors and the quality of the obtained mass spectra. By comparing the protein profiles of bacteria from patients not treated with antibiotics to those treated with antibiotics based on the presence/absence of specific signals and using the UniProt platform, it was possible to predict the probable mechanism of the action of the antibiotic used and the mechanism of drug resistance.

## 1. Introduction

Quick and reliable methods are needed to identify bacteria from post-operative wounds. The rapid identification of the pathogen causing the infection will enable to implement of an appropriate therapy [1]. Currently, the matrix-assisted laser desorption ionization time-of-flight mass spectrometry technique (MALDI-TOF MS) is used with great success [2]. The MALDI-TOF MS technique allows for quick and accurate identification of clinically relevant microorganisms [3,4].

Post-operative wound infection, also known as surgical site infections (SSIs), is a common healthcare problem. It is estimated that 2–7% of all operations develop SSIs, and 11% of all deaths in intensive care patients are related to post-operative wound infections [5]. Research confirms the incidence of SSIs (2.1%) and shows that infections occur even when surgeons take aseptic precautions during surgery and patients are treated before and after surgery [6]. Infections are associated with the bacterial contamination of the wound during surgery or later due to the deterioration of skin barriers during wound care [7].

The most common pathogens of hard to heal post-operative wound infections are *Escherichia coli*, *Staphylococcus aureus,* and *Enterococcus* spp. Other frequently identified microorganisms in infected post-operative wounds include *Pseudomonas aeruginosa*, *Klebsiella* spp., *Proteus* spp., *Citrobacter* spp. and coagulase-negative staphylococci [8,9]. Their role as infectious agents is due to the increasing number of highly virulent organisms that can survive in hospital conditions.

The widespread use of broad-spectrum antibiotics has likely led to the development of antibiotic-resistant and multi-drug-resistant strains, such as methicillin-resistant *S. aureus* (MRSA) and *P. aeruginosa* [10,11,12]. Antibiotic prophylaxis significantly reduces the risk of post-operative infections; however, there is still disagreement about the duration of the antibiotic therapy and the choice of antibiotics [13,14]. β-lactam antibiotics, quinolones, and lincosamide antibiotics are used in order to treat bacterial infections of post-operative wounds [15].

The MALDI-TOF MS is the simplest form of mass spectrometry, successfully applied to effectively detect bacteria in clinical samples [2]. The identification based on the MALDI-TOF MS consists of generating mass spectra from the analysis of whole cells or bacterial extracts representing mainly ribosomal proteins and other abundant bacterial proteins [16,17]. The obtained spectra are then matched with the references in the database. The identification accuracy depends on the appropriate quality of the spectrum and a close reference match in the database.

Some of the commercially available databases used to identify microorganisms are MALDI Biotyper (BrukerDaltonik GmbH, Bremen, Germany) and VITEK^®^MS (bioMérieux, Marcy l’Etoile, France) [18]. The quality of the protein profiles of the identified bacteria may be influenced by the types of matrix, the composition of the culture medium, and the temperature, pH, and incubation time of the bacterial cells with the method of sample preparation [19].

These changes are visible in the form of differences in the intensity and separation of individual signals and the loss or shift of signals, which may be caused by different expressions of the analysed proteins. In addition, an important factor is a procedure of sample application to the MALDI target plate and the selection of an appropriate matrix affecting the range of ionized molecules [20]. The MALDI-TOF MS can also be applied as an efficient tool to study the drug-resistance mechanism and the antibiotic drug metabolite identification [21,22].

The most frequently studied resistance is the one directed against β-lactam antibiotic drugs (cefotaxime, meropenem, piperacillin, tazobactam, penicillin, and amoxicillin), which inhibit the bacterial cell wall biosynthesis by affecting penicillin-binding proteins (PBPs) [23]. β-lactam resistance is a growing and disturbing phenomenon, and it mainly affects *Streptococcus pneumoniae*, *S. aureus*, and *P. aeruginosa*.

Bacterial resistance to β-lactams is determined by three main mechanisms: (I) the production of PBP proteins with low affinity for β-lactams while performing a catalytic function, (II) the regulation of β-lactam entry and efflux, and (III) the production of β-lactamases—enzymes hydrolysing β-lactam molecules [24]. Quinolone antibiotics, including ciprofloxacin, are the agents that inhibit DNA replication. The bactericidal activity is based on the inhibition of topoisomerase IV (Gram-positive bacteria) and topoisomerase II—DNA grainy (Gram-negative ones), enzymes indispensable for the course of replication and the provocative DNA twist [25]. Resistance to quinolone antibiotics has been a problem for over 40 years and maybe related to two mechanisms.

The first is related to chromosomal mutations in genes encoding the protein targets, or mutations causing reduced drug accumulation, either by a decreased uptake or by an increased efflux—they mainly concern *Enterobacteriaceae* bacteria, *P. aeruginosa*, and *Acinetobacter baumannii*. Acquired plasmid resistance genes that produce protective target proteins, drug modifying enzymes or drug efflux pumps represent the second mechanism of quinolone resistance (*Klebsiella pneumoniae*, *Enterobacter cloacae*, and *E. coli*) [26]. Lincosamide antibiotics, such as clindamycin inhibit bacterial protein synthesis by specific actions on the 50S subunit of the bacterial ribosome, possibly by influencing the initiation of the peptide chain. The mechanisms of resistance are similar to those of quinolone resistance and are characteristic of, among others, *Enterococcus* spp., *Streptococcus* spp., and *Staphylococcus* spp. [27].

The aim of the research was a new approach to development a spectrometric method to identify bacterial strains isolated from post-operative wounds of patients undergoing antibiotic therapy using the MALDI-TOF MS technique and the influence of culture conditions on the level of the identification and quality of mass spectra. Moreover, generated proteins profiles of strains derived from control patients (no antibiotic treatment) and those subjected to antibiotic therapy were compared based on the presence/absence of specific signals of bacterial molecular profile.

Then, the signals were analysed following their name and function by using the UniProt database as a pilot study. Utilization of the UniProt database was performed as an attempts to indicate the protein complexes responsible for molecular changes in bacterial profiles—untargeted analysis. Due to the use of MALDI-TOF MS technique and the UniProt database, we compared the obtained signals and, on this basis, it was possible to investigate whether there are factors contributing to the development of antibiotic resistance in isolates from patients undergoing antibiotic therapy.

## 2. Results and Discussion

### 2.1. Bacterial Identification

The sample preparation procedure and the MALDI-TOF MS analysis allowed us to obtain MS spectra and identify bacterial strains on post-operative wounds of patients. Bacterial strains were analysed in single-spectrum and MSP (Main spectra profiles) modes using the MALDI Biotyper 3.0 platform. Identification results and score values for both RAW and MSP spectra for each medium are summarized in Table 1.

The use of four culture media allowed for greater microbiological diversity. Due to this, it was possible to analyse the number of not only the species but also the strains colonizing difficult post-operative wounds.

Identifying the most significant number of bacterial species was possible through the use of non-selective media: BHI and MH, which allow the isolation of a wide variety of microorganisms. These media have been successfully used to isolate bacteria from clinical specimens [28,29]. Compared to other culture media, BHI was the only one that enabled the isolation of *Lactobacillus pentosus* and *Providenciastuartii*. Fewer bacterial species were identified on the selective BCP medium to isolate *Enterobacteriaceae* bacteria [30]. The BCP medium was the only one that allowed *Enterobacterkobei* to be detected. The smallest number of bacterial species involved in post-operative wound infections was isolated on the VRE medium, a selective medium for the culture of vancomycin-resistant enterococci, such as *E. faecalis*and *E. faecium* [31].

In summary, we obtained 75 different bacterial isolates, among which 71 were identified. Bacteria from 50 samples were identified with species confidence and 21 with genus confidence. The use of four different microbiological media allowed us to identify 34 strains belonging to 15 species and nine types of bacteria (Table 2). The highest percentage concerned bacteria of the genus *Enterococcus* (26.5%) and *Staphylococcus* (23.5%). *E. coli* accounted for 11% of all the identified bacteria. Each of the other types of bacteria accounted for <10% of all identified microorganisms.

The research by Gangania et al. and Akinkunmi et al., confirmed the high share of *E. coli* and *S. aureus* in post-operative wound infections; they also indicate *Staphylococcus*, *Enterococcus*, and *Streptococcus* bacteria. The research shows that *P. aeruginosa* also plays a role in surgical site infections [32,33]. The MALDI-TOF MS analysis did not allow the identification of three bacterial isolates (score < 1.7). This indicates the need to extend the currently available databases, also indicated by the literature data [34]. The insufficient number of mass spectra in the base of testers may also cause incorrect identification, which was proven by Body et al. [35].

As for bacterial species commonly found in clinical samples, the available databases are robust and sufficient to conclude that they are enough for the routine identification in clinical microbiology [36]. On the other hand, known human pathogens represent only 1% of the population of all microbial species in the world; therefore, the databases will not be fully complete [37]. Extending the databases will enable more accurate identification of microorganisms. For example, a group previously thought to be a single *Candida haemulonii* is now identified as a complex of species, three of which are regularly detected in clinical trials [38]. A similar situation applies to bacteria of the *Klebsiella* genus [39]. The newly discovered species belonging to these types of microorganisms appear to be of clinical importance. Updating the databases can solve the problem of misidentification or non-identification [40].

From the swabs taken from a patient treated with three antibiotics (B21) and two antibiotics, ciprofloxacin combined with metronidazole (B12), it was impossible to isolate the bacteria from any of the solid media used, which may indicate that the antibiotic therapy worked effectively. The presence of *Bacillus* spp. in the patient sample B21 may have been caused by the contamination of the sample.

The type of swabs used was also taken into account. It can be concluded that the analysis performed for liquid and solid swabs gave a similar number of identified bacteria at the species level.

### 2.2. Mass Spectra Analysis

**Figure 1:**
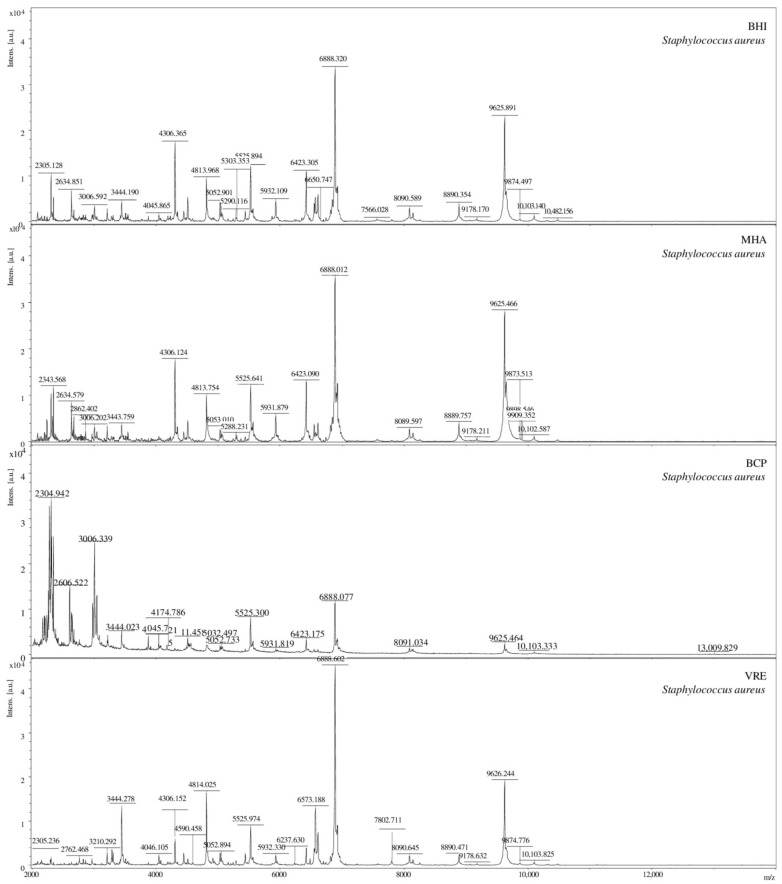
Comparison of the MS spectra for *S. aureus* bacteria isolated on four different culture media (BHI, BCP, MH, and VRE), [a.u.] = arbitrary units.

The analysis of mass spectra obtained for *S. aureus* bacteria isolated from each of the four culture media (Figure 1), for which score >2 showed that a different quality of the spectrum was obtained depending on the medium used. The highest identification score value was obtained for the bacteria isolated from the VRE medium (2.47) and BHI (2.35). The medium from which the bacteria giving the lowest identification scores were isolated was the selective BCP medium (2.03).

A salt blend added to the VRE medium and pigments in the BCP medium can reduce the yield or inhibit the analyte is ionized by blocking/suppressing the ionization process [41,42]. On the other hand, Złoch et al. investigated the effect of various solid culture media on the identification of *S. aureus* [43]. Their results showed that each medium allowed for the correct identification of microorganisms, and the differences between the score values were minimal.

**Figure 2:**
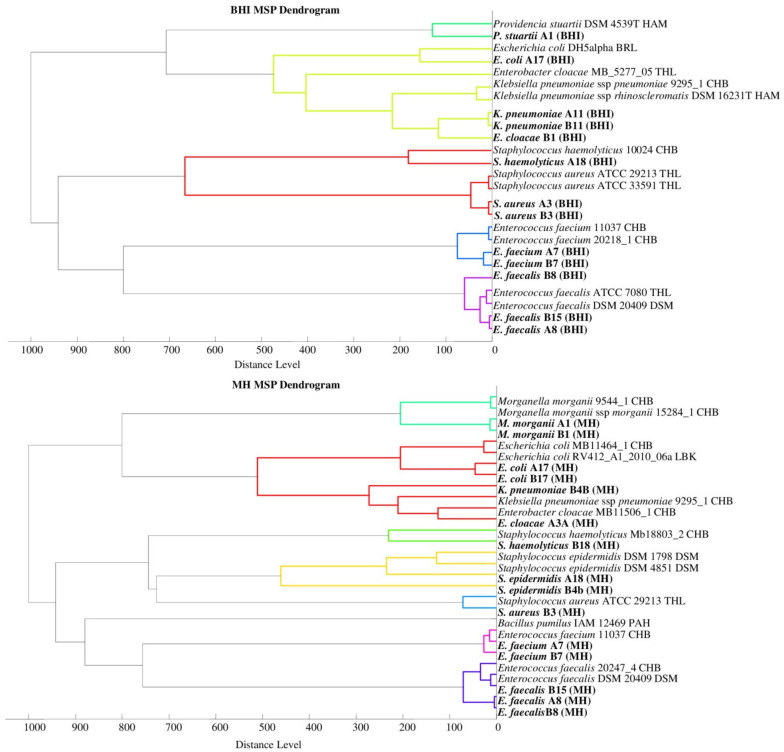

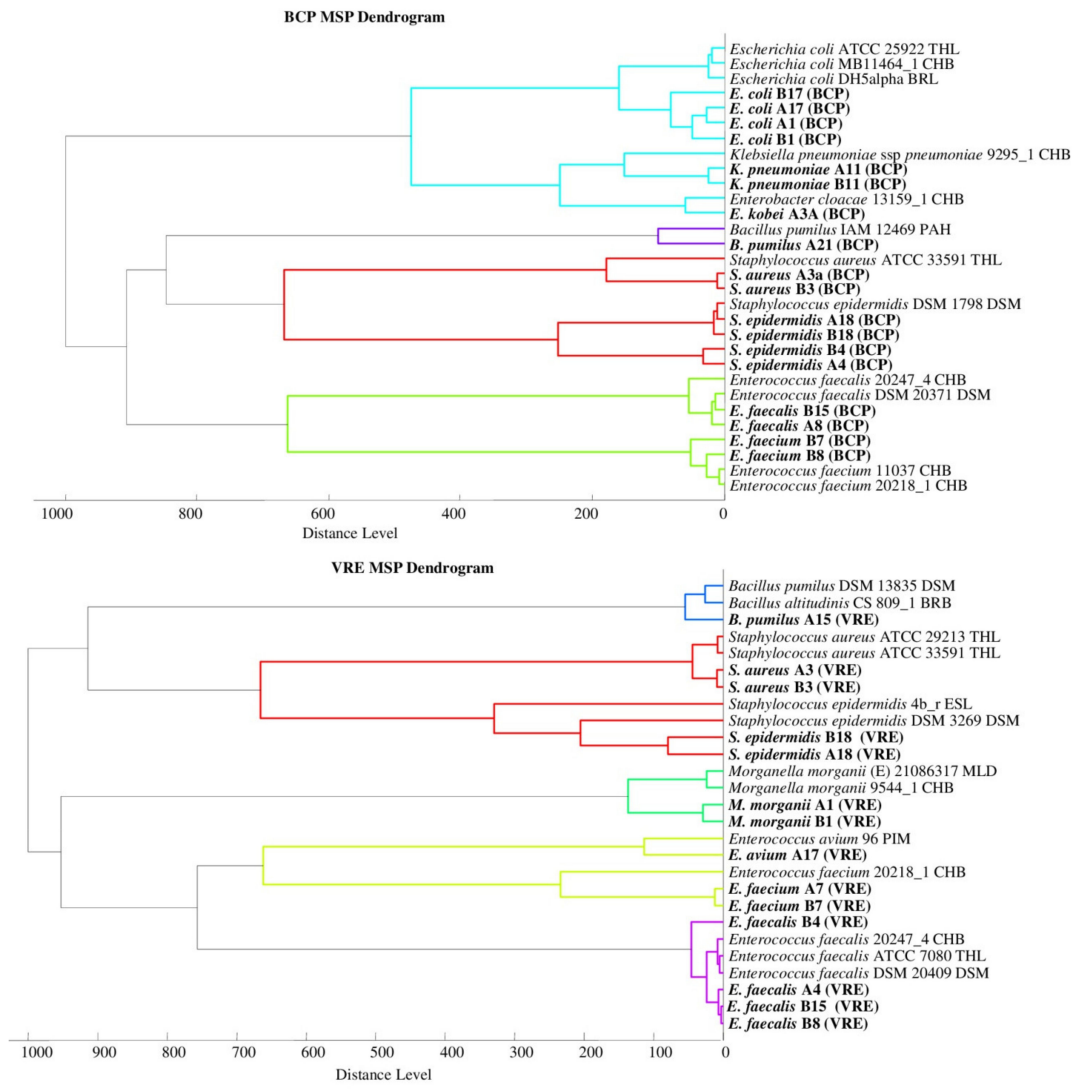
Phyloproteomic trees of the identified bacterial strains on all media and reference strains based on MSP identification via the MALDI Biotyper platform, MSP = main spectra.

Phyloproteomic relationships between isolates are presented in MSP dendrograms (Figure 2). All the phyloproteomic trees proved the relationship between the bacterial strains isolated from the biological material and the reference strains present in the database. Proteomic trees were made in order to confirm the correct identification of the isolated bacterial strain. Their analysis showed that five groups of bacteria were identified on the BHI medium, four groups on the BCP, five groups on the VRE, and eight groups on the MH. On each of the dendrograms of bacteria evolved from the BHI, BCP, and MH media, bacteria of the genus *Escherichia*, *Klebsiella,* and *Enterobacter* were grouped into one cluster may prove their close relationship.

The listed species of bacteria belong to the large *Enterobacteriaceae* family, and the results from the phyloproteomic tree correlate with their genetic background. This is confirmed, among other things, by the studies by Paradis et al. [44]. No close relationship was found for the bacteria of the genus *Staphylococcus* triggered on the MH medium as they were divided into three groups based on species (*S. haemolyticus*, *S. aureus*, and *S. epidermidis*). The analysis of phyloproteomic trees made for bacteria evolved from the remaining growth media (BHI, BCP, and VRE) allows us to conclude that there is a close relationship between these strains.

The analysis of the phylogenetic trees by Złoch et al. confirmed the relative origin of the *Staphylococcus* species [2]. The analysis of the VRE dendrogram shows that *Enterococcus* bacteria were divided into two groups (*E. Faecalis* and *E. faecium)*, and the genetic relationships presented by analogues phylogenetic trees were confirmed by the data obtained from the analysis of phyloproteomic trees [45].

### 2.3. Identification of Proteins with Specific Peak to the Predicted Antibiotic Mechanism of Action and Bacterial Drug-Resistance

Table 3 includes the specific peaks identified by the MALDI-TOF MS and their characteristics from the UniProt database. Moreover, the table does not include signals from uncharacterized proteins or proteins that are not associated with the action of antibiotics or antibiotic resistance. The values of *m/z* from the control sample and the test sample were compared. The samples tested were protein profiles obtained for *S. aureus* (patient treated with clindamycin), *E. cloacae* (patient treated with clindamycin) and *E. faecalis* (piperacillin combined with tazobactam).

Eighteen specific signals related to virulence, drug resistance, or the administered antibiotic’s mechanisms were identified for *S. aureus* /ATCC 29213 THL/. Among the signals appearing in the test sample, phenol-soluble modulin PSM-α-3 (*m/z* = 2656.8) can be distinguished. The studies of Queck et al. and Chatterjee et al. demonstrated that this protein is a rare example of combined antibiotic resistance and virulence factors in MRSA [46,47]. The increased PSM expression was shown to cause the increased virulence of the MRSA (methicillin-resistant *S. aureus)* and MDR (multidrug-resistant) *S. aureus* strains and contribute to the infection’s severity [65,66].

In addition, it was shown that PSM proteins are involved in the structuring of the bacterial biofilm of *S. aureus*, which entails the spread of infection and increases the resistance to antibiotics [67]. Generally, PSM toxin is a major virulence factor of *S. aureus* and primarily causes cytolysis in red and white blood cells. Among α-type phenol soluble modulins, the PSM-α3 plays the most prominent role in *S. aureus* virulence and, moreover, is the most cytotoxic member of the family [67].

The presence of the TraR/DksA family transcriptional regulation (*m/z* = 3006.2) on bacterial conjugation plasmids suggests their role in pathogenicity, virulence, and antibiotic resistance [48]. In the case of bacteria isolated from control group patients, the presence of antibiotic transport proteins (characteristic for the signal *m/z* = 6355.1), uncharacterized in the database protein synthesized in response to antibiotic use (*m/z* = 6589.8) and exported protein (*m/z* = 6743.1) was also demonstrated. These proteins can contribute to the development of antibiotic resistance [53].

Characteristic signals for immunoglobulin G building protein A (*m/z* = 9898.5) and protein A (*m/z* = 9909.4) being a virulence factor, especially in MRSA strains [55], have been reported. In addition, a signal characteristic of GCN5-related *N*-acetyltransferase (GNAT) was also identified, the presence of which was first confirmed in kanamycin and gentamycin resistant bacteria [54]. This result may indicate resistance to more than one antibacterial agent in the analysed *S. aureus* strain.

Compared with the control group, the test sample is mainly the signals characterized as 50S ribosomal protein L36 (*m/z* = 4290.7) and L33 (*m/z* = 5437.4 and 5872.7). The inhibition of the production of ribosomal proteins, thus, prevents the translation and biosynthesis of bacterial proteins [49]. Signals from various ABC transport proteins were also identified (*m/z* = 4365.1, 4379.9, 4822.6, 7022.9, and 8908.0). These proteins may affect the virulency of pathogenic strains and may be the target of antibiotics [50].

The lack of proteins from the ABC family will likely impair the import of many substances important for the life of bacterial cells and, thus, negatively affect their functioning [51]. Single-stranded DNA binding proteins (*m/z* = 4386.1) (ssDNA) (SSB), the signal of which does not appear only in the control sample, are necessary for the DNA replication and the protection of the delayed strand against the nuclease attack [52].

The patient who was identified with the presence of *E. cloacae*/MB_5277_05 THL/was treated with clindamycin. After using the antibiotic, a signal appeared corresponding to the Agmatinase protein (*m/z* = 3710.7; 5598.8), catalysing the reaction of agmatine hydrolysis to putrescine and urea. McCurtain et al. proved that the accumulation of agmatinase could result in the increased antibiotic tolerance by bacteria [58].

We found that the MS spectrum of bacteria after the administration of the antibiotic showed a signal from murein transglycosylase (*m/z* = 6417.9), responsible for breaking the glycosidic bond and for the catalysis of intramolecular and glycosylation [60]. These reactions can disintegrate the bacterial cell wall. On the other hand, the appearance of the BcsF cellulose biosynthesis protein was discovered (*m/z* = 7620.8), which is involved in the formation of a bacterial biofilm and, thus, increased the resistance to antibiotics [61].

Additionally, it can be assumed that the use of clindamycin may affect damage to the genetic material, because the mass spectrum showed a signal from the DNA polymerase V (*m/z* = 9410.181), which is part of the SOS response to the DNA damage [62]. The antibiotic administration likely caused dysregulation of the DNA transcription, which pretended the ParB partition protein signal (*m/z* = 10,315.8) and translation—the appearance of a factor favouring the hibernation of ribosomes HPF (*m/z* = 10,883.9) [63,64,68]. After the antibiotic treatment, the signal disappearance at *m/z* = 5571.8 for dehydrogenase (NADP (+)) was visible.

This protein is responsible, inter alia, for the binding of Mg^2+^ and Mn^2+^ ions; it has the activity of isocitrate dehydrogenase (NADP (+)) and takes part in the citric acid cycle [59]. On this basis, we concluded that this protein plays several important physiological roles, inter alia, in the reductive fatty acid biosynthesis, proper oxidation–reduction balance as well as in oxidative damage. Moreover, the lack of it in a bacterial cell may result in a disturbance of the cell metabolism and may have a negative impact on the integrity of cell membranes [59].

Patients with identified *E. faecalis*/ATCC 7080 THL/were treated with piperacillin combined with tazobactam. Both of the used drugs belong to the group of β-lactam antibiotics that inhibit the biosynthesis of the bacterial cell wall. A significant change in the mass spectrum of bacteria from the control sample, compared to the test group, is the appearance of the attenuator leader peptide (*m/z* = 2443.3) in the latter, which is a response to the action of the applied antibiotics [56]. The disappearance of the signal characteristic of the 50S ribosomal protein L30 (*m/z* = 6341.0) was demonstrated. This can result in disturbances in translation and protein synthesis [57].

The conducted pilot research indicates the possibility of using the UniProt database to the preliminary match of the proteins contained therein with the spectra generated by the MS, including predicting the mechanisms of the action of antibiotics and the mechanisms of the antibiotic resistance of bacteria. Notably, the products of antibiotic metabolism can also be biologically active substances [69]. To confirm the obtained results, it is necessary to perform additional analysis by the utilisation of, e.g., shotgun techniques, such as liquid chromatography with tandem mass spectrometry (LC-MS/MS) or combined sodium dodecyl sulphate–polyacrylamide gel electrophoresis (SDS-PAGE) and MALDI-TOF/TOF MS methods [70,71].

### 2.4. Selection of Sample Preparation Conditions to Determine Microorganisms Using the MALDI-TOF MS Technique

Table 4 compares the obtained identification indicators of the tested bacterial species in terms of the liquid medium used, the method of sample preparation, and the incubation time. Empty spaces in the table mean that it was not possible to obtain mass spectra from these samples.

Both methods used allowed for the correct species identification at a similar level. The analysis of the obtained results showed that it was possible to correctly identify *E. faecalis* after 4 h of incubation using both media and both methods of sample preparation. The use of the same conditions and a 6-h incubation allowed for the identification at the species level of *S. aureus*. Identification of *E. coli* was possible only after the application of liquid TSB medium.

The standard method turned out to be a better method of preparing bacterial extracts for MALDI-TOF MS analysis compared with the Sepsityper method. This conclusion confirms Drevinek et al.’s research, which proved that the technique of using ethanol for pre-treatment and formic acid in combination with acetonitrile for the preparation of bacterial extracts showed the highest extraction efficiency and spectral quality. Researchers found this method universal for samples suspected of having Gram (+) or Gram (−) bacteria [72]. The research by Burckhardt et al. proved that 20 h of incubation was sufficient to detect multidrug-resistant bacteria and MRSA strains [73].

Pomastowski et al. showed that shortening the incubation time to 12 h enabled the generation of good-quality spectra and the correct identification of microorganisms. Their research showed that different incubation time intervals affected the quality of the recorded spectra; yet, they did not detect any significant qualitative changes in the peak patterns—they only observed an increase in the intensity (100% with 24-h incubation) [17]. The research results by van den Bijllaardt et al. suggested that 10 h of incubation of bacterial cells during tests related to reading sensitivity to antibiotics was sufficient [74]. Altun et al., on the other hand, obtained results that showed that a 5.5-h incubation of bacteria from blood cultures enabled the satisfactory identification of bacteria using MALDI-TOF MS and the Vitek 2 database [75].

Oviaño et al., in their research, proved that using the MALDI-TOF MS technique and the sonication-based extraction method enabled the precise identification of bacteria in the liquid bacterial culture in 15 min [76]. Haiko et al. used the method of the urine short incubation MALDI-TOF for the rapid identification of urinary pathogens [77]. They correctly identified 86% of G (−) bacteria; however, this method did not identify G (+) bacteria. Due to this approach, it was possible to shorten the analysis time to 4–6 h compared to the 24 h required by conventional methods.

Shortening the analysis time will significantly speed up microbiological diagnostics, which will additionally enable a faster application of the appropriate therapy for saving the patient’s health and life. Additionally, Gajdács et al. proved that the MALDI-TOF MS technique compared to conventional biochemical methods showed higher efficiency in identifying anaerobic bacteria, such as *Cutibacterium* spp., *Bacteroides/Parabacteroides* spp., and *Clostridium* spp. in blood samples [78].

**Figure 3:**
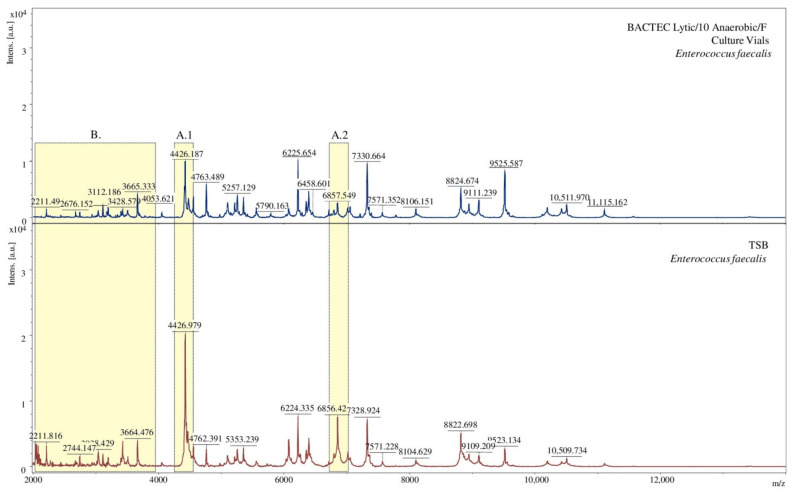
Comparison of MALDI-TOF MS mass spectra in terms of the medium used, [a.u.] = arbitrary units. (**A.1**), (**A.2**) show signals of different intensity; (**B**) denotes spectral fragments differing in the number of signals.

The obtained mass spectra were also analysed for every tested bacteria. Below is a comparison of spectra under specific conditions for exemplary bacterial species. The spectra obtained for bacteria isolated from two different media slightly differ from each other. Some of the signals for the TSB medium vary in intensity (Figure 3(A.1,A.2)). Additionally, due to using the BACTEC Lytic/10 Anaerobic/F Vial Culture Media, a more significant number of signals were obtained (Figure 3B).

**Figure 4:**
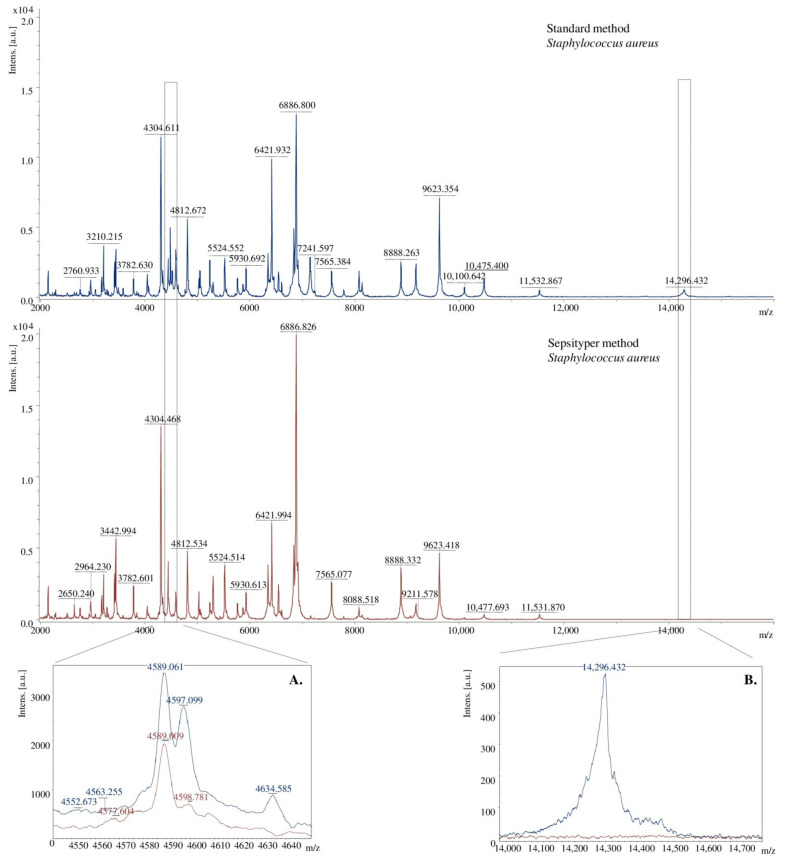
Comparison of the MALDI TOF MS mass spectra in terms of the method of the preparation of bacterial extracts, [a.u.] = arbitrary units. (**A**)–more split signals were obtained for the standard method, (**B**)–more signals were obtained for the standard method.

Using two methods of preparing bacterial extracts, spectra of different quality were obtained. The signals received for samples prepared with the Sepsityper method had a higher intensity than those equipped with the standard method. On the other hand, the second method allowed obtaining more separated signals (Figure 4A) and more of them (Figure 4B). The differences may result from the different buffer composition of the analysed sample.

## 3. Experimental

### 3.1. Chemicals and Reagents

The reagents of high purity were used. Brain Heart Infusion Agar (BHI), Mueller Hilton Agar (MH), Glucose Bromocresol Purple Agar (BCP), Vancomycin Resistance Enterococci Agar Base (VRE), Tryptic Soy Broth (TSB) (all from Sigma-Aldrich, Steinheim, Germany), and BACTEC Lytic/10 Anaerobic/F Vial Culture Media (BD, Franklin Lakes, NJ, USA) were used to isolate bacteria and to culture peptone water.

To obtain the bacterial protein extract, HPLC-grade water, formic acid (FA), acetonitrile (ACN), ethanol, trifluoroacetic acid (TFA) (Sigma-Aldrich, Steinheim, Germany) were applied. In order to prepare samples, in addition to the reagents mentioned before, a MBT Sepsityper IVD Kit was used (BrukerDaltonik GmbH, Bremen, Germany). The kit contained Lysis Buffer and Washing Buffer. For the MALDI-TOF MS analysis, α-cyano-4-hydroxycinnamic acid (HCCA) (Sigma-Aldrich, Schaffhausen, Switzerland) and Bacterial Test Standard (BTS) (BrukerDaltonik GmbH, Bremen, Germany) were used.

### 3.2. Patient and Sample Characterization

Biological material was the superficial swab from post-operative wounds from fifteen patients with SSIs following abdominal surgery of the Provincial Polyclinic Hospital in Toruń. Thirteen patients were treated with antibiotics administered through an intravenous infusion. Six patients received one antibiotic: cefotaxime (B1), clindamycin (two patient—B2, B17), meropenem (B4), ciprofloxacin (B11), and metronidazole (16), while the remaining patients received a combination of the antibiotic treatment with piperacillin and tazobactam (three patients—B7, B15, and B18), ciprofloxacin and metronidazole (two patients—B8 and B12), penicillin and clindamycin (B9) and clindamycin, metronidazole, and amoxicillin (B21). The other two patients were not treated with an antibiotic (control samples).

Before smearing, the post-operative wounds were washed with a sterile physiological saline solution. Two swabs were then collected from each patient. The first was performed with a dry swab stick, which was then placed in Amies liquid transport medium (DeltaSwab Amies, Barcelona, Spain) (swab sample “b”). The second swab was taken with a swab stick from a tube with Amies solid transport medium (BorPol, Gliwice, Poland) (swab sample “a”).

The research was approved by the Bioethics Committee of Nicolaus Copernicus University in Toruń (decision no. 585/2017). All the study participants were informed orally and in writing about the purpose of the study, completed the questionnaire and a declaration of their voluntary consent to participate in the study.

### 3.3. Isolation of Post-Operative Wound Bacteria

Swab stick “a” was placed in 1 mL of the transport medium, and 1 mL of the transport medium was added to the swab sample “b”. 100 μL of the obtained suspension was transferred onto the four growth media: two non-selective (BHI, MH) and two selective (VRE, BCP) and incubated for 24 h at 37 °C. After the incubation, to receive pure bacterial colonies, streaking was made and followed by incubation again under the same conditions as previously [79]. Protein extracts were made from pure bacterial colonies according to standard acetonitrile-formic acid extraction protocol [2].

### 3.4. MALDI-TOF MS Analysis

For the MALDI-TOF MS analysis, bacterial protein extracts from the obtained pure bacterial cultures were prepared based on the Bruker guideline with slight modifications (standard method). Approximately10 mg of bacterial colonies were added to 150 μL of ultrapure water and mixed thoroughly. Subsequently, 300 μL of absolute EtOH was added to the resulting suspension, and then thoroughly vortexed and centrifuged (15871 RCF, 5 min).

After that, the supernatant was discarded, and the pellet was dried in a vacuum concentrator at 37 °C for 7–10 min. We added 1–12 μL FA to the dried pellet in proportion to the amount of biological material and carefully mixed by pipetting. Next, the same volume of ACN was added, mixed and centrifuged (15871 RCF, 5 min). Afterwards, 1 μL of each sample was transferred onto MALDI MTP 384 polished steel target sample spot (BrukerDaltonik GmbH, Bremen, Germany) in duplicate.

After air-drying, the sample spots were overlaid with HCCA matrix dissolved in standard solvent (50% water, 47.5% ACN, and 2.5% TFA) at the final concentration of 10 mg/mL. The MALDI target plate with samples was analysed using the ultrafleXtreme MALDI-TOF/TOF mass spectrometer (BrukerDaltonik GmbH, Bremen, Germany) equipped with smart beam-II laser-positive mode according to the procedure described by Pomastowski et al. [80].

The calibration analysis was originally performed with a Bruker Bacterial Test Standard (BTS). The spectra were collected manually using the flexControl software with parameters *m/z* range: 2000–20,000, acceleration voltage at 25 kV, global attenuator offset at 20%, attenuator offset at 34%, range at 34%, laser power at 40%, and 500 shots in-one-single spectra to frequency 2500. The obtained spectra were smoothed and brought to the baseline.

Validated mass spectra were processed with the use of the software provided by the manufacturer–flexControl and flexAnalysis and, subsequently, used for the bacterial identification via the MALDI Biotyper Compass platform (BrukerDaltonik GmbH, Bremen, Germany) based on both raw spectra (RAW) and Main Spectra (MSP) according to the manufacturer’s protocol. Based on the MSPs, phyloproteomic dendrograms were generated to verify the correct identification of the isolated microorganisms by their relationship with the reference strains and the relationship between the isolated strains. The UniProt platform was used to predict the mechanism of the action of the antibiotic administered to the patient on the bacterial cell based on the obtained *m/z* values.

### 3.5. Selection of Sample Preparation Conditions to Determine Microorganisms with MALDI-TOF MS

Two Gram-positive strains (*S. aureus*BHI.B3a, *E. faecalis* VRE.B3a) and one Gram-negative (*E. coli* BHI.B4a) bacterial strain were selected for further analysis. From one-day cultures, one inoculation loop was transferred to a glass tube with 2 mL peptone water to obtain a bacterial suspension with a density equal to 0.5 McFarland. This process was performed in duplicate. Then, 100 μL of suspension was transferred to 9.9 mL liquid growth media TSB, vortexed and diluted 1:1 (*v/v*) in TSB. From the second tubes, 100 μL inoculum was transferred to 900 μL peptone water and mixed thoroughly.

Next, 200 μL of suspension was added to the vial with BACTEC Lytic/10 Anaerobic/F culture media using a syringe with a needle and mixed. Both of the bacterial cultures were incubated for 4, 6, and 24 h at 37 °C. After each incubation time, the bacterial extracts were prepared according to the previously described standard method and using the MBT Sepsityper IVD Kit according to the protocol provided by the manufacturer. We added 200 μL of Lysis Buffer to a 1 mL of sample and vortexed. The sample was centrifuged (18407 RCF, 2 min), the supernatant was discarded, and 1 mL of Washing Buffer was added, mixed thoroughly and centrifuged again (18407 RCF, 1 min).

The supernatant was discarded, and the pellet was dried at 37 °C using a vacuum concentrator (LABCONCO CentriVap DNA^®^Concentrator, Labconco Corporation, Kansas City, MO, USA). After that, FA and ACN were added proportionally to the amount of the dried pellet. The MALDI-TOF MS analysis was performed as previously described. For each bacterial species, 12 samples were prepared, including two liquid media, three different incubation times, and two sample preparation methods (six samples for TSB medium for bacteria incubated consecutively 4, 6, and 24 h, and extracts prepared by the standard and the Sepsityper method). In total, 36 samples were analysed.

## 4. Conclusions

Using the MALDI-TOF MS approach, we confirmed the high involvement of *Staphylococcus*, *Enterococcus*, and *Escherichia* bacteria in the process of inducing post-operative wound infections. The conducted research proved that the selection of the culture medium and the preparation of bacterial extracts significantly affected the identification factor and the quality of the mass spectra obtained. The best quality of the spectra was obtained using non-selective media and the standard method of sample preparation, which is also confirmed by the literature data.

The use of non-selective media, such as the BHI or MH, also leads to obtaining more types of bacteria. The incubation time strongly influenced the bacterial identification; therefore, we concluded that the optimal time can significantly accelerate the microbiological diagnosis of post-operative wound infections and implement the individual therapy faster. The use of the UniProt database made it possible to indicate proteins influencing the emergence of drug resistance and predict the antibiotics’ actions.

The obtained results of the studies showed that MALDI-TOF MS with the use of additional databases, such as UniProt, can be a relevant tool for the detection of antibiotic resistance. Microbiological proteomics is a powerful tool for basic microbiological research not only in determining microbial mechanisms and physiology but also as a clinical diagnosis and antimicrobial therapy guideline. The obtained results may enable us to verify the medical diagnosis; they bring hope for the development of methods enabling a faster diagnosis due to the detection of disease changes at the cellular level prior to the occurrence of clinical changes, the monitoring of the course of the disease treatment, and the development of drugs for individual patients.

## Figures and Tables

**Table 1:** MALDI-TOF MS identification results from raw samples (primary identification, RAW) and main spectra library (second identification, MSP) with the level of the identification of bacteria strains isolated from solid medium (non-selective: BHI, MH and selective: BCP, VRE) using the Biotyper 3.0 platform.

**Non-Selective Growth Media**									
**BHI**					**MH**				
**Sample** **Name**	**RAW**		**MSP**		**Sample** **Name**	**RAW**		**MSP**	
	**Best Match**	**Score Value**	**Best Match**	**Score Value**		**Best Match**	**Score Value**	**Best Match**	**Score Value**
B1a	*Providencia stuartii* DSM 4539T HAM	2.10	*Providencia stuartii* DSM 4539T HAM	1.99	B1a	*Morganella morganii* ssp *morganii* 15284_1 CHB	2.04	*Morganella morganii* ssp *morganii* 15284_1 CHB	2.05
B1b	*Enterobacter cloacae* MB_5277_05 THL	1.91	*Enterobacter cloacae* MB_5277_05 THL	1.78	B1b	*Morganella morganii* 9544_1 CHB	1.93	*Morganella morganii* 9544_1 CHB	1.93
B3a	*Staphylococcus aureus* ATCC 25923 THL	2.35	*Staphylococcus aureus* ATCC 33591 THL	2.26	B3aD	*Enterobacter cloacae* MB_5277_05 THL	2.03	*Enterobacter cloacae* MB11506_1 CHB	1.99
B3b	*Staphylococcus aureus* ATCC 25923 THL	2.19	*Staphylococcus aureus* ATCC 29213 THL	1.99	B3aM	*Staphylococcus aureus* ATCC 33591 THL	2.15	*Staphylococcus aureus* ATCC 29213 THL	2.12
B4a	*Staphylococcus epidermidis* 10547 CHB	2.28	-	1.69	B3b	*Staphylococcus aureus* ATCC 33591 THL	2.23	*Staphylococcus aureus* ATCC 29213 THL	2.19
B4b	*Staphylococcus epidermidis* ATCC 14990T THL	1.90	-	1.53	B4a	*Staphylococcus epidermidis* 10547 CHB	1.99	-	1.51
B7a	*Enterococcus faecium* 20218_1 CHB	2.14	*Enterococcus faecium* 20218_1 CHB	2.15	B4bD	*Klebsiella pneumoniae* ssp *pneumoniae* DSM 30104T HAM	2.13	*Klebsiella pneumoniae* *ssp pneumoniae* 9295_1 CHB	1.90
B7b	*Enterococcus faecium* 11037 CHB	2.09	*Enterococcus faecium* 11037 CHB	2.09	B4bM	*Staphylococcus epidermidis* 0547 CHB	1.98	*Staphylococcus epidermidis* DSM 4851 DSM	1.74
B8a	*Enterococcus faecalis* ATCC 7080 THL	2.19	*Enterococcus faecalis* ATCC 7080 THL	2.14	B7a	*Enterococcus faecium* 11037 CHB	2.25	*Enterococcus faecium* 11037 CHB	2.25
B8b	*Enterococcus faecalis* DSM 2570 DSM	2.18	*Enterococcus faecalis* DSM 20409 DSM	1.91	B7b	*Enterococcus faecium* 11037 CHB	2.35	*Enterococcus faecium* 11037 CHB	2.29
B9a	-	1.45	-	1.39	B8a	*Enterococcus faecalis* DSM 20409 DSM	2.10	*Enterococcus faecalis* DSM 20409 DSM	2.03
B11a	*Klebsiella pneumoniae* ssp *ozaenae* DSM 16358T HAM	2.15	*Klebsiella pneumoniae* ssp *rhinoscleromatis* DSM 16231T HAM	2.06	B8b	*Enterococcus faecalis* ATCC 7080 THL	2.12	*Enterococcus faecalis* 20247_4 CHB	1.91
B11b	*Klebsiella pneumoniae* ssp *pneumoniae* DSM 30104T HAM	2.07	*Klebsiella pneumoniae* ssp *pneumoniae* 9295_1 CHB	2.04	B11b	*Klebsiella pneumoniae* ssp *pneumoniae* 9295_1 CHB	2.32	*Klebsiella pneumoniae* ssp *pneumoniae* 9295_1 CHB	2.32
B15a	-	1.39	-	1.33	B15a	*Bacillus pumilus* IAM 12469 PAH	1.74	-	1.64
B15b	*Enterococcus faecalis* ATCC 7080 THL	2.30	*Enterococcus faecalis* DSM 20409 DSM	2.26	B15b	*Enterococcus faecalis* ATCC 7080 THL	2.43	*Enterococcus faecalis* DSM 20409 DSM	2.35
B17a	*Escherichia coli* ATCC 25922 THL	2.25	*Escherichia coli* DH5alpha BRL	2.18	B17a	*Escherichia coli* MB11464_1 CHB	1.99	*Escherichia coli* MB11464_1 CHB	1.88
B18a	*Staphylococcus haemolyticus* 10024 CHB	2.23	*Staphylococcus haemolyticus* 10024 CHB	1.97	B17b	*Escherichia coli* RV412_A1_2010_06a LBK	1.95	*Escherichia coli* RV412_A1_2010_06a LBK	1.95
B18b	*Staphylococcus epidermidis* CCM 4505 CCM	2.10	-	1.68	B18a	*Staphylococcus epidermidis* DSM 1798 DSM	1.85	*Staphylococcus epidermidis* DSM 1798 DSM	1.89
B21a	*Bacillus pumilus* IAM 12469 PAH	1.77	-	1.68	B18b	*Staphylococcus haemolyticus* Mb18803_2 CHB	2.01	*Staphylococcus haemolyticus* Mb18803_2 CHB	1.93
B21a (white)	*Lactobacillus pentosus* DSM 20199 DSM	1.82	-	1.21	B21a	-	1.68	-	1.59
**Selective Growth Media**									
**BCP**					**VRE**				
**Sample** **Name**	**RAW**		**MSP**		**Sample** **name**	**RAW**		**MSP**	
	**Best Match**	**Score Value**	**Best Match**	**Score Value**		**Best Match**	**Score Value**	**Best Match**	**Score Value**
B1a	*Escherichia coli* ATCC 25922 THL	2.22	*Escherichia coli* ATCC 25922 THL	2.09	B1a	*Morganella morgani* 9544_1 CHB	1.96	*Morganella morganii* 9544_1 CHB	1.80
B1b	*Escherichia coli* MB11464_1 CHB	2.15	*Escherichia coli* MB11464_1 CHB	2.10	B1b	*Morganella morgani* (E) 21086317 MLD	1.99	*Morganella morganii* (E) 21086317 MLD	1.89
B3aD	*Enterobacter kobei* DSM 13645T DSM	2.20	*Enterobacter cloacae* 13159_1 CHB	2.08	B3a	*Staphylococcus aureus* ATCC 29213 THL	2.47	*Staphylococcus aureus* ATCC 29213 THL	2.21
B3aM	*Staphylococcus aureus* ATCC 33591 THL	2.03	*Staphylococcus aureus* ATCC 33591 THL	1.88	B3b	*Staphylococcus aureus* ATCC 29213 THL	2.47	*Staphylococcus aureus* ATCC 33591 THL	2.25
B3b	*Staphylococcus aureus* ATCC 33591 THL	1.95	*Staphylococcus aureus* ATCC 33591 THL	1.84	B4a	*Enterococcus faecalis* ATCC 7080 THL	2.34	*Enterococcus faecalis* DSM 20409 DSM	2.33
B4a	*Staphylococcus epidermidis* ATCC 14990T THL	2.04	*Staphylococcus epidermidis* DSM 1798 DSM	1.84	B4b	*Enterococcus faecalis* ATCC 7080 THL	2.06	*Enterococcus faecalis* ATCC 7080 THL	1.83
B4b	*Staphylococcus epidermidis* ATCC 14990T THL	2.14	*Staphylococcus epidermidis* DSM 1798 DSM	1.84	B7a	*Enterococcus faecium* 20218_1 CHB	1.97	*Enterococcus faecium* 20218_1 CHB	1.95
B7a	*Enterococcus faecium* 11037 CHB	2.19	*Enterococcus faecium* 20218_1 CHB	2.15	B7b	*Enterococcus faecium* 20218_1 CHB	1.95	*Enterococcus faecium* 20218_1 CHB	1.95
B7b	*Enterococcus faecium* 11037 CHB	2.05	*Enterococcus faecium* 20218_1 CHB	2.09	B8b	*Enterococcus faecalis* ATCC 7080 THL	2.20	*Enterococcus faecalis* ATCC 7080 THL	2.15
B8a	*Enterococcus faecalis* DSM 20371 DSM	1.98	*Enterococcus faecalis* DSM 20371 DSM	1.90	B15a	*Bacillus pumilus* DSM 13835 DSM	2.09	*Bacillus pumilus* DSM 13835 DSM	2.09
B8b	*Enterococcus faecium* 11037 CHB	2.32	*Enterococcus faecium* 11037 CHB	2.38	B15b	*Enterococcus faecalis* ATCC 29212 CHB	2.20	*Enterococcus faecalis* 20247_4 CHB	2.11
B11a	*Klebsiella pneumoniae* ssp *pneumoniae* 9295_1 CHB	2.22	*Klebsiella pneumoniae* ssp *pneumoniae* 9295_1 CHB	2.17	B17a	*Enterococcus avium* 96 PIM	1.87	*Enterococcus avium* 96 PIM	1.87
B11b	*Klebsiella pneumoniae* ssp *pneumoniae* 9295_1 CHB	2.10	*Klebsiella pneumoniae* ssp *pneumoniae* 9295_1 CHB	2.10	B18a	*Staphylococcus epidermidis* 10547 CHB	2.16	*Staphylococcus epidermidis* DSM 3269 DSM	1.92
B15a	-	1.61	-	1.61	B18b	*Staphylococcus epidermidis* 10547 CHB	1.92	*Staphylococcus epidermidis* 4b_r ESL	1.87
B15b	*Enterococcus faecalis* 20247_4 CHB	2.29	*Enterococcus faecalis* 20247_4 CHB	2.18	B21a	*Bacillus altitudis* CS 809_1 BRB	1.72	-	1.57
B17a	*Escherichia coli* DH5alphaBRL	2.19	*Escherichia coli* DH5alpha BRL	2.01					
B17b	*Escherichia coli* DH5alpha BRL	2.08	*Escherichia coli* MB11464_1 CHB	1.84					
B18a	*Staphylococcus epidermidis* 10547 CHB	2.08	*Staphylococcus epidermidis* DSM 1798 DSM	1.94					
B18b	*Staphylococcus epidermidis* DSM 1798 DSM	2.00	*Staphylococcus epidermidis* DSM 1798 DSM	2.13					
B21a	*Bacillus pumilus* IAM 12469 PAH	1.73	-	1.68					

The probability of correct identification in the MALDI Biotyper 3.0 system was expressed in the form of a point index and a graphical index: 2.300–3.000: reliable identification of the microorganism up to species level; 2.000–2.299: reliable identification of the microorganism to genus level and the probable result of identification to species level; 1.700–1.999: indicates the probable result of identification to the level of the genus; ≤1.699: not reliable identification result.; BHI–Brain Heart Infusion Agar, MH–Mueller Hinton Agar, BCP–Glucose Bromocresol Purple Agar, VRE–Vancomycin Resistance Enterococci Agar Base; a–swab stick from a tube with Amies solid transport medium (BorPol, Gliwice, Poland); b–dry swab stick placed in Amies liquid transport medium (DeltaSwab Amies, Barcelona, Spain); D–large colony, and M–small colony.

**Table 2:** Bacterial strains isolated from swabs from post-operative wounds and identified in biological material.

Bacteria from Biological Material	Reference Bacteria Strain
*Bacillus*	
*B. altitudis* VRE.B21a	*B. altitudis* CS 809_1 BRB
*B. pumilus* BCP.B21a	*B. pumilus* IAM 12469 PAH
*B. pumilus* VRE.B15a	*B. pumilus* DSM 13835 DSM
*Enterobacter*	
*E. cloacae* BHI.B1b	*E. cloacae* MB_5277_05 THL
*E. kobei* BCP.B3aD	*E. kobei* DSM 13645T DSM
*Enterococcus*	
*E. avium* VRE.B17a	*E. avium* 96 PIM
*E. faecalis* BCP.B8a	*E. faecalis* DSM 20371 DSM
*E. faecalis* BCP.B15b	*E. faecalis* 20247_4 CHB
*E. faecalis* BHI.B15b	*E. faecalis* ATCC 7080 THL
*E. faecalis* BHI.B8b	*E. faecalis* DSM 2570 DSM
*E. faecalis* VRE.B15b	*E. faecalis* ATCC 29212 CHB
*E. faecalis* MHA.B8a	*E. faecalis* DSM 20409 DSM
*E. faecium* BHI.B7b	*E. faecium* 11037 CHB
*E. faecium* BHI.B7a	*E. faecium* 20218_1 CHB
*Escherichia*	
*E. coli* BHI.B17a	*E. coli* ATCC 25922 THL
*E. coli* BCP.B1b	*E. coli* MB11464_1 CHB
*E. coli* BCP.B17a	*E. coli* DH5alpha BRL
*E. coli* MHA.B17a	*E. coli* RV412_A1_2010_06a LBK
*Klebsiella*	
*K. pneumoniae* BCP.B11a	*K. pneumoniae* ssp *pneumoniae* 9295_1 CHB
*K. pneumoniae* BHI.B11a	*K. pneumoniae* ssp *ozaenae* DSM 16358T HAM
*K.pneumoniae* BHI.B11b	*K.pneumoniae* ssp *pneumoniae* DSM 30104T HAM
*Lactobacillus*	
*L. pentosus* BHI.B21a(white)	*L. pentosus* DSM 20199 DSM
*Morganella*	
*M. morgani* MHA.B1b	*M. morgani* 9544_1 CHB
*M. morgani* VRE.B1b	*M. morgani* (E) 21086317 MLD
*M. morganii* MHA.B1a	*M. morganii* ssp *morganii* 15284_1 CHB
*Providencia*	
*P. stuartii* BHI.B1a	*P. stuartii* DSM 4539T HAM
*Staphylococcus*	
*S. aureus* BCP.B3aM	*S. aureus* ATCC_33591_THL
*S. aureus* BHI.B3b	*S. aureus* ATCC_25923_THL
*S. epidermidis* BCP.B4a	*S. epidermidis* ATCC 14990T THL
*S. epidermidis* BHI.B4a	*S. epidermidis* 10547 CHB
*S. epidermidis* BCP.B18b	*S. epidermidis* DSM 1798 DSM
*S. epidermidis* BHI. B18b	*S. epidermidis* CCM 4505 CCM
*S. haemolyticus* BHI.B18a	*S. haemolyticus* 10024 CHB
*S. haemolyticus* MHA.B18b	*S. haemolyticus* Mb18803_2 CHB

**Table 3:** Summary of signals observed on MALDI-TOF MS spectra with characterized *m/z* values, according to the database of Universal Protein (UniProt).

***S. aureus /*** **ATCC 29213 THL/(Antibiotic: Clindamycin)**					
***m/z***	**Control Sample**	**Test Sample**	**Protein**	**Putative Function**	**Ref.**
2656.8	-	+	Phenol-soluble modulin PSM-alpha-3	virulence factor	[46,47]
3006.2	-	+	TraR/DksA family transcriptional regulator	virulence, antibiotic resistance, pathogenesis	[48]
4290.7	+	-	50S ribosomal protein L36	builds ribosome, translation	[49]
4365.1	+	-	ABC transporter ATP-binding protein		[50,51]
4379.9	+	-	Teichoic acid ABC transporter permease		[50,51]
4386.1	+	-	Single-stranded DNA-binding protein	DNA replication	[52]
4822.6	+	-	Oligopeptide ABC superfamily ATP binding cassette transporter. binding protein (Protein CysJ)		[50,51]
5437.4	+	-	50S ribosomal protein L33.	builds ribosome, translation	[49]
5872.7	+	-	50S ribosomal protein L33	builds ribosome, translation	[49]
6355.1	-	+	Antibiotic transport-associated protein		[53]
6589.8	-	+	Antibiotic resistance protein		[53]
6743.1	-	+	Exported protein		[53]
7022.9	+	-	ABC transporter ATP-binding protein		[50,51]
8111.7	-	+	GCN5-related *N*-acetyltransferase (GNAT)	kanamycin and gentamycin resistance	[54]
8908.0	+	-	Compound ABC uptake transporter ATP-binding protein.		[50,51]
9898.5	-	+	Immunoglobulin G binding protein A	binding protein A	[55]
9909.4	-	+	Protein A	pathogenic factor	[55]
***E. faecalis /*** **ATCC 7080 THL/(antibiotics: piperacylin and tazobactam)**					
***m/z***	**Control sample**	**Test sample**	**Protein**	**Putative Function**	
2443.3	‒	+	Attenuator leader peptide	response to antibiotic	[56]
6341.0	+	‒	50S ribosomal protein L30	builds ribosome, translation	[57]
***E. cloacae /*** **MB_5277_05 THL/(antibiotic: clindamycin)**					
***m/z***	**Control sample**	**Test sample**	**Protein**	**Putative Function**	
3710.7	‒	+	Agmatinase	catalysis of the reaction: agmatine + H_2_O = putrescine + urea	[58]
5571.8	+	‒	Isocitrate dehydrogenase (NADP(+))	cofactor binding sites Mg^2+^, Mn^2+^;	[59]
5598.8	‒	+	Agmatinase	catalysis of the reaction: agmatine + H_2_O = putrescine + urea	[58]
6417.9	‒	+	Murein transglycosylase	break the glycosidic bond	[60]
7620.8	‒	+	Cellulose biosynthesis protein BcsF	necessary for biofilm formation	[61]
9410.2	‒	+	DNA polymerase V	part of the SOS response to DNA damage	[62]
10,315.8	+	‒	ParB partition protein	regulation of transcription	[63]
10,883.9	‒	+	Ribosome hibernation promoting factor HPF	primary metabolic process, regulation of translation	[64]

The control and test sample columns are highlighted as green and orange, respectively, to differentiate between them.

**Table 4:** Comparison of the obtained identification indices of the tested bacterial species in terms of the applied liquid culture media, methods of sample preparation, and incubation time.

Bacteria Species	Time	Standard Method		Sepsityper Method	
		Vials	TSB	Vials	TSB
*S. aureus*	4 h	1.85–1.97		1.79–1.88	2.05–2.16
	6 h	2.38–2.42	2.31–2.39	2.36–2.39	2.25–2.30
	24 h	2.25–2.34	2.24–2.33	2.30–2.34	2.31–2.42
*E. coli*	4 h	-	2.35–2.37	-	2.31–2.38
	6 h	-	2.31–2.33	-	2.20–2.28
	24 h	-	2.27–2.32	-	2.10–2.17
*E. faecalis*	4 h	2.33–2.35	2.26–2.36	2.28–2.32	2.06–2.18
	6 h	2.00–2.02	1.76–1.88	2.02–2.06	
	24 h	2.39–2.42	2.45–2.47	2.19–2.24	

2.300–3.000: reliable identification of the microorganism up to species level (green); 2.000–2.299: reliable identification of the microorganism to genus level and probable result of identification to species level (green); 1.700–1.999: indicates probable result of identification to the level of the genus (yellow); ≤1.699: not reliable identification result (red). TSB–Tryptic Soy Broth; and Vials–BACTEC Lytic/10 Anaerobic/F.

## Data Availability

Not applicable.

## Sample Availability

Samples of the compounds (antibiotics) and tissue samples are available from the authors.
